# Graphical analysis of multi-environmental trials for wheat grain yield based on GGE-biplot analysis under diverse sowing dates

**DOI:** 10.1186/s12870-023-04197-9

**Published:** 2023-04-17

**Authors:** Fatemeh Saeidnia, Majid Taherian, Seyed Mahmoud Nazeri

**Affiliations:** Agricultural and Horticultural Science Research Department, Khorasan Razavi Agricultural and Natural Resources Research and Education Center, Agricultural Research, Education and Extension Organization, Mashhad, 91769-83641 Iran

**Keywords:** Adaptability; GE interaction; GGE biplot; grain yield; heritability, sowing date; stability; wheat

## Abstract

**Background:**

Information on the nature and extent of genetic and genotype × environment (GE) interaction is extremely rare in wheat varieties under different sowing dates. In the present study, the GGE biplot method was conducted to investigate genotype × environment interaction effects and evaluate the adaptability and yield stability of 13 wheat varieties across eight sowing dates, in order to facilitate comparison among varieties and sowing dates and identify suitable varieties for the future breeding studies.

**Results:**

Considerable genotypic variation was observed among genotypes for all of the evaluated traits, demonstrating that selection for these traits would be successful. Low broad sense heritability obtained for grain yield showed that, both genetic and non-genetic gene actions played a role in the control of this trait, and suggested that indirect selection based on its components which had high heritability and high correlation with yield, would be more effective to improve grain yield in this germplasm. Hence, selection based on an index may be more useful for improvement of this trait in recurrent selection programs. The results of the stability analysis showed that the environmental effect was a major source of variation, which captured 72.21% of total variation, whereas G and GE explained 6.94% and 18.33%, respectively. The partitioning of GGE through GGE biplot analysis showed that, the first two PCs accounted for 54.64% and 35.15% of the GGE sum of squares respectively, capturing a total of 89.79% variation. According to the GGE biplot, among the studied varieties, the performance of Gascogen was the least stable, whereas Sirvan, Roshan, and Pishtaz had superior performance under all sowing dates, suggesting that they have a broad adaptation to the diverse sowing dates. These varieties may be recommended for genetic improvement of wheat with a high degree of adaptation.

**Conclusion:**

The results obtained in this study demonstrated the efficiency of the GGE biplot technique for selecting high yielding and stable varieties across sowing dates.

## Background

Wheat is one of the most important staple-foods with global production of over 700 million tones and supplies about 20% of the total calories and daily proteins to 4.5 billion people worldwide [1, 2]. Due to its international trade volume being greater than all other major food crops combined [3], wheat occupies a central place in human nutrition and plays an important role in the national economy of developing countries. To satisfy the increasing food demand of the growing world population, accessing high production through improving wheat yields is the demand of the twenty-first century, because the arable land area will not increase beyond current levels [4].

Sowing date is an important management factor in the production of any crop [5], because different sowing dates cause the vegetative and reproductive stages of the plant to encounter the different temperatures, solar radiation, and day length, and thereby, it affects the growth and development of plants. Based on climatic conditions, there is a suitable sowing date for each region, which is determined by weather conditions, availability of land, moisture, seeds, the desired variety, and the probable time for the spread of pests and diseases. Cultivating wheat at the wrong time, either sooner or later, has many adverse effects [6]; so that the delay in the sowing date leads to the decrease in the potential performance due to the lack of timely establishment of the plant, lack of sufficient growth before facing the autumn cold and also the lack of receiving a part of the available solar radiation by the plant shader due to the reduction in the length of the growth period of plants. Early planting can lead to yield reduction, because longer wheat plant life in the field increases the possibility of diseases and adverse consequences related to grain yield [7]. Inversely, wheat cultivation at the proper time, leads to high germination percentage, good tillering, timely phenological growth, and production of strong plants with a strong root system, reduction of dormancy, increase of seed weight for all growth types, and plant survival. Flowers et al. [8], in a study on the effect of sowing date on yield and yield components of two wheat cultivars reported that sowing date had a great effect on the wheat yield, and a delay in sowing date reduced wheat yield by 24%. Subhan et al. [9] in a study on the effect of sowing dates on wheat reported that, the delay in sowing date has the greatest effect among the yield components on the thousand grain weight of wheat. Therefore, according to the importance of sowing dates on crop yield, this study was carried out on different varieties of bread wheat on eight different sowing dates, in order to achieve optimal sowing dates in our region.

The genotype × environment (GE) interaction results from genotypic rank fluctuation or fluctuations in the absolute differences between genotypes without rank change [10, 11]. GEI usually hinders the accuracy of yield estimation, reduces the association of genotypic and phenotypic values, and complicates the process of selecting genotypes with superior performance [12]. Therefore, knowing the magnitude of GE interactions is crucial for the development of high-yielding and stable cultivars over a wide range of environments [13]. Consequently, to evaluate the relative performance of genotypes over the test environments for the development of high-yielding and stable varieties, plant breeders apply multi-environment trials (METs) [14].

Univariate linear regression models [15] and multivariate models of Additive Main effects and Multiplicative Interactions (AMMI) [16] and Genotype × Genotype-Environment interaction (GGE) biplot [17] have been used to study and interpret G × E interaction. Among these methods, the GGE biplot is more interpretative and has been identified as an innovative methodology for the analysis and visualization of the pattern of GEI in multi-environment studies [18], and has been recognized as a preferred tools in mega environment analysis, evaluation of genotypes, association of traits, and heterotic pattern analysis [19, 20]. In recent literature, the application of AMMI analysis and GGE biplot analysis for visualization and interpretation of multi-environment experiment data have been widely discussed [21, 22]. Yan et al. [23] indicated that the GGE biplot was superior to the AMMI biplot in mega-environment analysis and genotype evaluation.

The efficiency of a selection program is mainly dependent on the nature and extent of genetic variability and heritability of the traits [13]. At this juncture, information about statistical parameters such as heritability, G × E interactions, correlations among various traits, and predicted and observed genetic gain through selection, greatly helps to devise and implement an appropriate breeding program [13]. Moreover, grain yield in grasses is the result of a complex combination of many variables that affect plant growth throughout the growing period. Therefore, direct selection is not always effective in improving it. Appropriate models for indirect selection that can predict yield and define the ideal genotype, is more effective [24, 25]. Information about the association between yield and its related traits can be used to improve the efficiency of breeding programs by identifying suitable indicators for selecting superior genotypes [25]. However, appropriate traits used for the development of the proper models for indirect selection should have significant genetic variability.

The GE interaction reduces the association of phenotypic and genotypic values and selection progress, and results in bias in the estimations of gene effects and combining ability of different traits that are sensitive to environmental changes [26]. Knowledge of GE interaction can help plant breeders to reduce the cost of genotype evaluation through eliminating unnecessary testing locations [21]. Information pertaining to genetic and GE interactions effects using wheat varieties grown on different sowing dates is lacking. Based on our knowledge, it is the first report on the application of the GGE biplot method for the analysis of genotype × sowing dates interaction in wheat. Information obtained from this study can facilitate selection of ecologically adapted and genetically diverse plant materials that are stable at different sowing dates. In this context, the present study is an attempt to (i) assess genetic diversity and estimate variance components and heritability of grain yield and its components in wheat varieties; (ii) clarify the most important components that are critical to the grain yield; (iii) visually evaluate the adaptability and grain yield stability of 13 wheat varieties across eight sowing dates based on the GGE biplot, in order to facilitate visual comparison among varieties and sowing dates; (v) identify varieties that have similar response pattern over all sowing dates, as well as, high yield for future breeding programs using GGE biplot.

## Results

### Analysis of variance and genetic analysis

Results from the combined ANOVA revealed that, sowing date had significant effect on all traits. There was significant difference (*P* ˂ 0.01) between the genotypes in terms of all traits; indicating considerable genotypic variation among the selected varieties (Table 1). Genotype × sowing date (G × S) interaction was also significant for all traits; which shows the different response of varieties to sowing dates in terms of these traits. The interaction of year × sowing date (Y × S) was significant for grain yield (GY) and biological yield (BY); indicating different performance of sowing dates in each year of the experiment and refers to the differences in the climatic conditions of each year.

**Table 1:** Combined analysis of variance for measured traits in 13 varieties of wheat in different sowing dates during three years (2012–2014)

Source of variation	Year (Y)	Replication /Y	Sowing date (S)	Y × S	S × rep /Y	Genotype (G)	S × G	Y × G	Y × S × G	Error
d.f	2	6	7	14	42	12	24	84	168	576
Traits	Mean squares									
GY	26.10^**^	1.03^n.s^	781.10^**^	2.75^**^	0.85^n.s^	36.60^**^	10.57^**^	14.80^**^	0.26^n.s^	0.33
BY	9.40^n.s^	5.43^n.s^	2437.32^**^	15.20^**^	1.27^n.s^	368.03^**^	13.28^**^	75.70^**^	1.30^n.s^	0.87
NSP	107,685.01^n.s^	46,289.00^n.s^	5,152,470.26^**^	33,164.45^n.s^	51,718.50^n.s^	899,893.32^**^	13,407.50^**^	329,864.08^**^	1670.35^n.s^	61,208.44
NGSP	391.60^n.s^	81.63^n.s^	15,328.08^**^	14.05^n.s^	28.72^n.s^	1667.53^**^	114.90^**^	361.50^**^	9.50^n.s^	9.80
GWSP	0.98^**^	0.06^n.s^	34.59^**^	0.03^n.s^	0.04^n.s^	22.30^**^	10.03^**^	0.57^**^	0.03^n.s^	0.02
TGW	2.40^n.s^	13.77^n.s^	10,314.73^**^	86.40^n.s^	2.80^n.s^	1113.25^**^	24.58^**^	401.17^**^	15.70^**^	3.20
HI	0.062^**^	0.012^n.s^	1.900^**^	0.004^n.s^	0.003^n.s^	0.211^**^	0.030^**^	0.044^**^	0.002^n.s^	0.001

*BY* Biological yield, *GWSP* Grain weight per spike, *GY* Grain yield, *HI* Harvest index, *NGSP* Number of grains per spike, *NSP* Number of spikes per m^2^, *TGW* Thousand grain weight

^**^show significance at the 0.01 probability level

n.s: not significant

Mean comparisons of wheat varieties for the evaluated traits based on the average of three years are given in Table 2. The mean grain yield (GY) ranged from 2.78 to 5.11 t/ha. Among the studied varieties, Pishgam, Pishtaz, and Mihan had the higher values of GY; while, Behrang produced the lowest grain yield. The biological yield (BY) varied considerably and ranged from 10.97 to 17.79 t/ha. The highest value of this trait was obtained for Roshan, and the lower values were detected for Gascogen and Behrang. The higher values of thousand seed weight (TSW) were obtained for Sirvan and Parsi, and the lowest value was detected for Bezostaya. The varieties of Falat, Sirvan, and Baz showed higher values in the harvest index, respectively. Meanwhile, the lowest value of this trait was obtained in Bezostaya (Table 2). Moreover, among the sowing dates, 6th November showed the highest values of all evaluated traits; and the lowest values of all traits were obtained on 6th April. Another noteworthy point in Table 2 is that the results of mean comparisons for three years of study verified exactly the results which were obtained from combined ANOVA about the effect of years on evaluated traits.

**Table 2:** Mean comparisons of years, varieties, and sowing dates for measured traits in wheat during three years (2012–2014)

**Year (Y)**	GY (t/ha)	BY (t/ha)	NSP	NGSP	TGW (g)	HI (%)
Y1	4.44 a	13.95 a	461.39 a	24.48 a	35.53 a	27.79 ab
Y2	3.98 b	13.67 a	431.25 a	23.21 a	35.62 a	26.07 b
Y3	4.49 a	13.97 a	463.86 a	25.36 a	35.74 a	28.72 a
**Varieties**						
Pishgam	5.11 a	13.78 c	497.00 c	26.63 d	33.53 f	30.87 c
Gascogen	3.73 f	10.97 e	364.70 k	20.42 f	29.87 h	23.75 g
Falat	4.56 c	12.91 d	459.90 f	29.24 a	34.46 e	33.14 a
Chamran	4.36 de	13.45 c	442.31 g	26.69 d	35.95 d	0.14 d
Roshan	3.49 g	17.79 a	618.72 a	16.70 g	36.81 c	18.45 h
Parsi	4.75 b	14.54 b	472.36 e	27.04 c	39.82 a	30.08 d
Pishtaz	4.99 a	14.29 b	484.28 d	28.42 b	39.14 b	32.24 b
Sirvan	4.42 cd	13.03 d	422.79 i	29.06 a	40.13 a	32.78 ab
Mihan	5.05 a	14.19 b	511.14 b	26.51 d	31.64 g	29.39 e
Oroom	4.23 e	12.84 d	419.53 j	23.18 e	33.42 f	26.43 f
Bezostaya	3.05 h	12.99 d	346.62 l	14.05 h	28.93 i	16.18 i
Behrang	2.78 i	11.12 e	293.75 m	20.48 f	39.01 b	23.19 g
Baz	4.40 d	12.83 d	440.22 h	28.63 b	34.65 e	32.51 ab
**Sowing dates**						
6th September	3.52 e	15.43 d	391.34 f	19.71 f	41.00 b	21.63 e
6th October	7.11 b	17.73 b	657.76 b	35.28 b	44.36 a	40.26 a
6th November	7.79 a	19.43 a	720.38 a	35.54 a	44.39 a	40.58 a
6th December	5.88 c	16.02 c	545.92 c	33.24 c	40.58 b	37.39 b
6th January	3.87 d	12.76 e	520.19 d	27.01 d	33.82 d	30.23 c
6th February	3.62 e	13.06 e	393.77 e	24.61 e	34.74 c	27.52 d
6th March	2.26 f	11.13 f	282.70 g	15.23 g	27.86 e	17.37 f
6th April	0.37 g	5.32 g	101.64 h	3.96 h	18.27 f	05.08 g

Means followed by the same letters in each column and each treatment are not significantly different according to the LSR test at the 5% level of probability

*BY* Biological yield, *GY* Grain yield, *HI* Harvest index, *NGSP* Number of grains per spike, *NSP* Number of spikes per m^2^, *TGW* Thousand grain weight

Phenotypic and genotypic coefficients of variation (PCV and GCV) are presented in Table 3. Phenotypic coefficient of variation had a range of 0.21% for HI to 25.17% for NSP. Genotypic coefficient of variation ranged from 0.17% for HI to 19.83% for NSP. The estimates of broad-sense heritability and variance components for all traits are displayed in Table 3. For BY, NGSP, GWSP, and HI, the estimates of genotype variance (σ^2^_g_) had the highest portion of phenotypic variance; while, for GY and TGW, the genotype × year (σ^2^_gy_) effect had the highest components of phenotypic variance. Based on the combined data over three years and eight sowing dates, broad-sense heritability estimates ranged from 31.39% for GY to 76.17% for BY. Moreover, the heritability of yield components (i.e. NSP, NGSP, GWSP, and TGW) were higher than that of grain yield (Table 3).

**Table 3:** Genetic parameters including variance components, broad-sense heritability (h^2^_b_), phenotypic coefficient of variation (PCV), and genetic coefficient of variation (GCV) of measured traits in 13 varieties of wheat in eight sowing dates during 2012–2014

Traits	Variance components				h^2^_b_ (%)	GCV (%)	PCV (%)
	σ^2^_g_	σ^2^_gy_	σ^2^_e_	σ^2^_p_			
GY	0.16	0.61	0.33	0.51	31.39	12.01	16.89
BY	3.89	3.10	0.87	5.11	76.17	14.68	16.82
NSP	7754.06	13,674.74	61,208.44	12,498.52	62.04	19.83	25.17
NGSP	16.68	14.67	9.80	23.16	72.00	16.74	19.73
GWSP	0.1629	0.0225	0.0200	0.3097	52.60	13.17	16.92
TGW	9.77	16.06	3.20	15.46	63.17	8.88	11.18
HI	0.0019	0.0018	0.0010	0.0029	65.88	0.17	0.21

*σ*^*2*^_*g*_ genotype variance; *σ*^*2*^_*gy*_ genotype × year variance, *σ*^*2*^_*e*_ error variance; *σ*^*2*^_*p*_ phenotypic variance; *h*^*2*^_*b*_ broad-sense heritability, *GCV* Genetic coefficient of variation, *PCV* Phenotypic coefficient of variation, *BY* Biological yield, *GWSP* Grain weight per spike, *GY* Grain yield, *HI* Harvest index, *NGSP* Number of grains per spike, *NSP* Number of spikes per m^2^, *TGW* Thousand grain weight

The stepwise multiple linear regression method was applied to determine the variables accounting for the majority of grain yield variation (Table 4). Results showed that under early sowing dates, the number of spikes (NSP) was the most important component of grain yield (partial *R* ^2^ = 89–97%); while, under late sowing dates, the number of grains per spike (NGSP) was the most important component (partial *R* ^2^ = 74–92%). Moreover, under all of the sowing dates, NSP, NGSP, TGW, and BY were identified as effective yield components and explained over 90% of the observed variation for grain yield (Table 4).

**Table 4:** Results from stepwise regression analysis for predicting grain yield of wheat varieties evaluated in eight sowing dates

Sowing dates	Variables entered	Parameter estimate	Partial R^2^	Model R^2^	F
6^th^ September	NSP	0.0129	0.9701	0.9701	357.46^**^
	HI	15.8781	0.0222	0.9924	29.19^**^
	TGW	0.0619	0.0042	0.9966	11.09^**^
	NGSP	-0.1507	0.0013	0.9979	5.17^*^
	BY	-0.1201	0.0014	0.9993	13.78^**^
	Intercept				100.05^**^
6^th^ October	NSP	0.0050	0.9633	0.9633	289.10^**^
	NGSP	0.0587	0.0126	0.9759	5.24^*^
	TGW	0.0454	0.0169	0.9928	21.25^**^
	BY	0.1771	0.0023	0.9952	3.86^n.s^
	HI	7.1900	0.0030	0.9982	11.87^*^
	Intercept	-6.3423			95.25^**^
6^th^ November	NSP	0.0041	0.9455	0.9455	190.87^**^
	NGSP	0.1021	0.0480	0.9935	73.96^**^
	BY	0.2263	0.0045	0.9980	20.90^**^
	HI	6.5005	0.0011	0.9992	10.90^*^
	Intercept	-5.8101			101.81^**^
6^th^ December	NSP	0.0096	0.8970	0.8970	95.79^**^
	NGSP	0.0649	0.0911	0.9881	76.47^**^
	Intercept	-1.5232			34.38^**^
6^th^ January	BY	0.3202	0.9976	0.9976	70.17^**^
	NSP	0.0016	0.0019	0.9995	21.35^**^
	HI	10.5209	0.0000	0.9995	31.02^**^
	TGW	0.0047	0.0003	0.9998	8.32^*^
	Intercept	-4.1575			1040.74^**^
6^th^ February	NGSP	0.1369	0.9266	0.9266	138.79^**^
	BY	0.1605	0.0673	0.9939	110.63^**^
	Intercept	-1.8553			83.92^**^
6^th^ March	NGSP	0.0822	0.9243	0.9243	109.86^**^
	NSP	0.0064	0.0703	0.9946	103.73^**^
	TGW	0.0207	0.0026	0.9972	6.55^*^
	Intercept	-1.6423			38.71^**^
6^th^ April	NGSP	0.0756	0.7407	0.7407	14.28^*^
	BY	0.0833	0.2558	0.9964	286.55^**^
	Intercept	-0.5402			213.16^**^

*BY* Biological yield, *GY* Grain yield, *HI* Harvest index, *NGSP* Number of grains per spike, *NSP* Number of spikes per m^2^, *TGW* Thousand grain weight

^*^ and ^**^ significant at the 0.05 and 0.01 probability levels, respectively

n.s: not significant

The correlation coefficients were partitioned into direct and indirect effects through path analysis (Table 5). Results showed that, under early sowing dates, HI and NSP had higher positive direct effects on grain yield. The higher positive indirect effects on grain yield were observed for NSP via NGSP, BY, and TGW, under different early sowing dates. Under late sowing dates, NGSP had the highest positive direct effects on grain yield. The higher positive indirect effects were detected for NGSP via TGW, BY, and NSP (Table 5).

**Table 5:** Direct and indirect effects of grain yield components in wheat varieties evaluated in eight sowing dates

Sowing dates	Traits	Direct effect	Indirect effect via					Total correlation with GY
			X1	X2	X3	X4	X5	
6^th^ September	BY (X1)	0.20	-	0.12	0.02	0.12	0.17	0.63
	HI (X2)	0.76	0.27	-	-0.002	-0.76	0.69	0.96
	TGW (X3)	0.10	0.009	-0.0003	-	-0.0004	-0.0044	0.10
	NGSP (X4)	-0.64	0.403	0.64	0.003	-	0.56	0.96
	NSP (X5)	0.05	0.98	-0.97	-0.05	0.97	-	0.98
	Residual	0.03						
6^th^ October	BY (X1)	0.38	-	-0.07	0.16	-0.05	0.29	0.71
	HI (X2)	0.28	-0.16	-	0.11	0.20	0.13	0.56
	TGW (X3)	0.10	0.04	-0.04	-	-0.04	0.03	0.09
	NGSP (X4)	0.17	-0.02	0.22	-0.06	-	0.28	0.59
	NSP (X5)	0.47	0.36	-0.22	-0.02	0.24	-	0.83
	Residual	0.04						
6^th^ November	BY (X1)	0.42	-	-0.09	-0.09	0.29		0.53
	HI (X2)	0.27	-0.06	-	0.27	0.14		0.62
	NGSP (X3)	0.33	-0.07	0.33	-	0.18		0.77
	NSP (X4)	0.36	0.25	0.18	0.18	-		0.97
	Residual	0.03						
6^th^ December	NGSP (X1)	0.49	-	0.34				0.83
	NSP (X2)	0.51	0.44	-				0.95
	Residual	0.11						
6^th^ January	BY (X1)	0.30	-	0.11	-0.08	-0.03		0.30
	HI (X2)	0.34	0.03	-	0.09	0.30		0.76
	TGW (X3)	0.02	-0.013	0.003	-	0.007		0.02
	NSP (X4)	0.13	-0.02	0.28	0.34	-		0.73
	Residual	0.02						
6^th^ February	BY (X1)	0.26	-	0.17				0.43
	NGSP (X2)	0.79	0.17	-				0.96
	Residual	0.08						
6^th^ March	TGW (X1)	0.25	-	0.20	0.22			0.67
	NGSP (X2)	0.43	0.22	-	0.33			0.98
	NSP (X3)	0.37	0.27	0.30	-			0.94
	Residual	0.10						
6^th^ April	BY (X1)	0.49	-	0.46				0.95
	NGSP (X2)	0.51	0.46	-				0.97
	Residual	0.19						

*BY* Biological yield, *GY* Grain yield, *HI* Harvest index, *NGSP* Number of grains per spike, *NSP* Number of spikes per m^2^, *TGW* Thousand grain weight

### Stability analysis

**Fig. 1:**
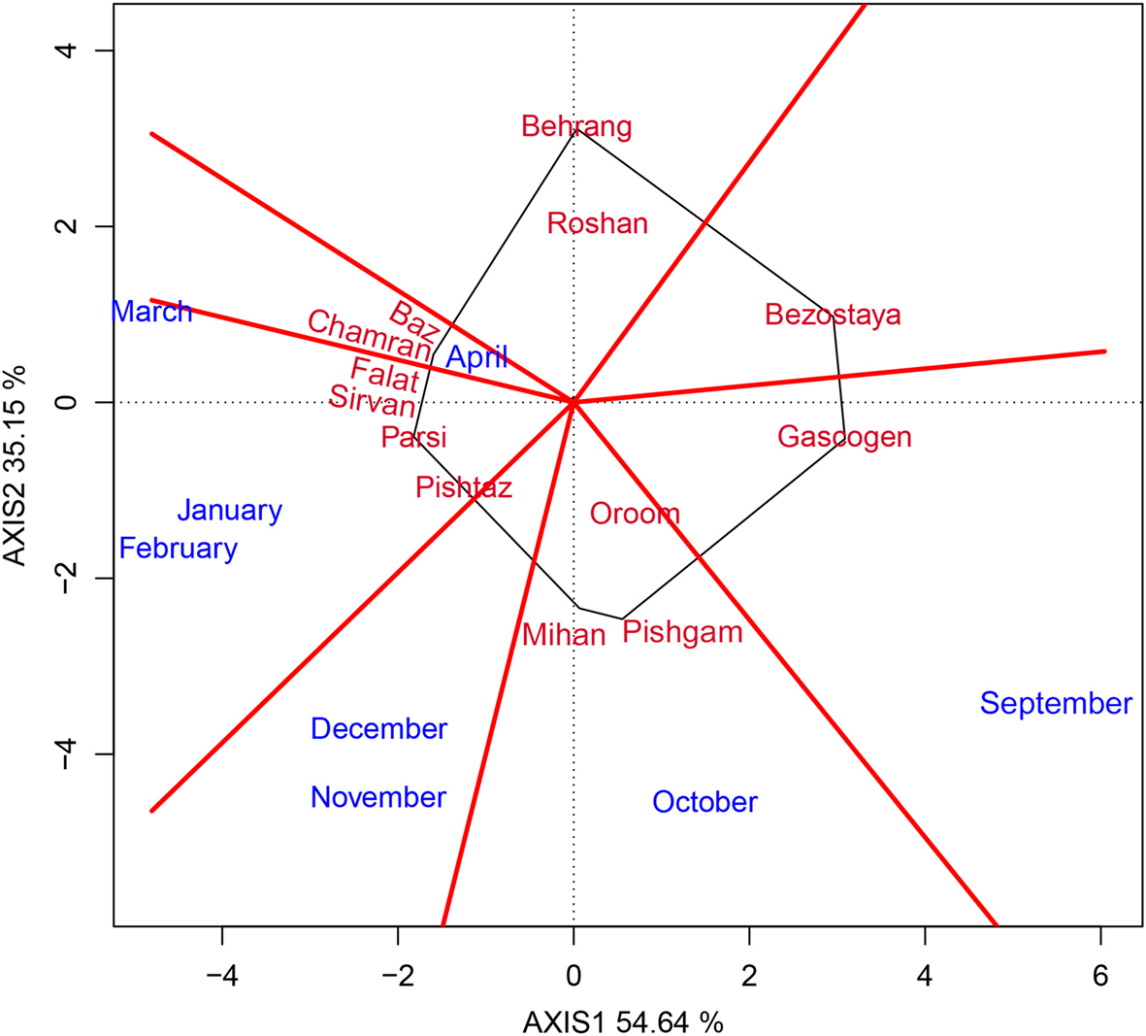
‘Polygon’ view of the GGE biplot to show which variety of wheat performed better in which sowing date in terms of grain yield

**Fig. 2:**
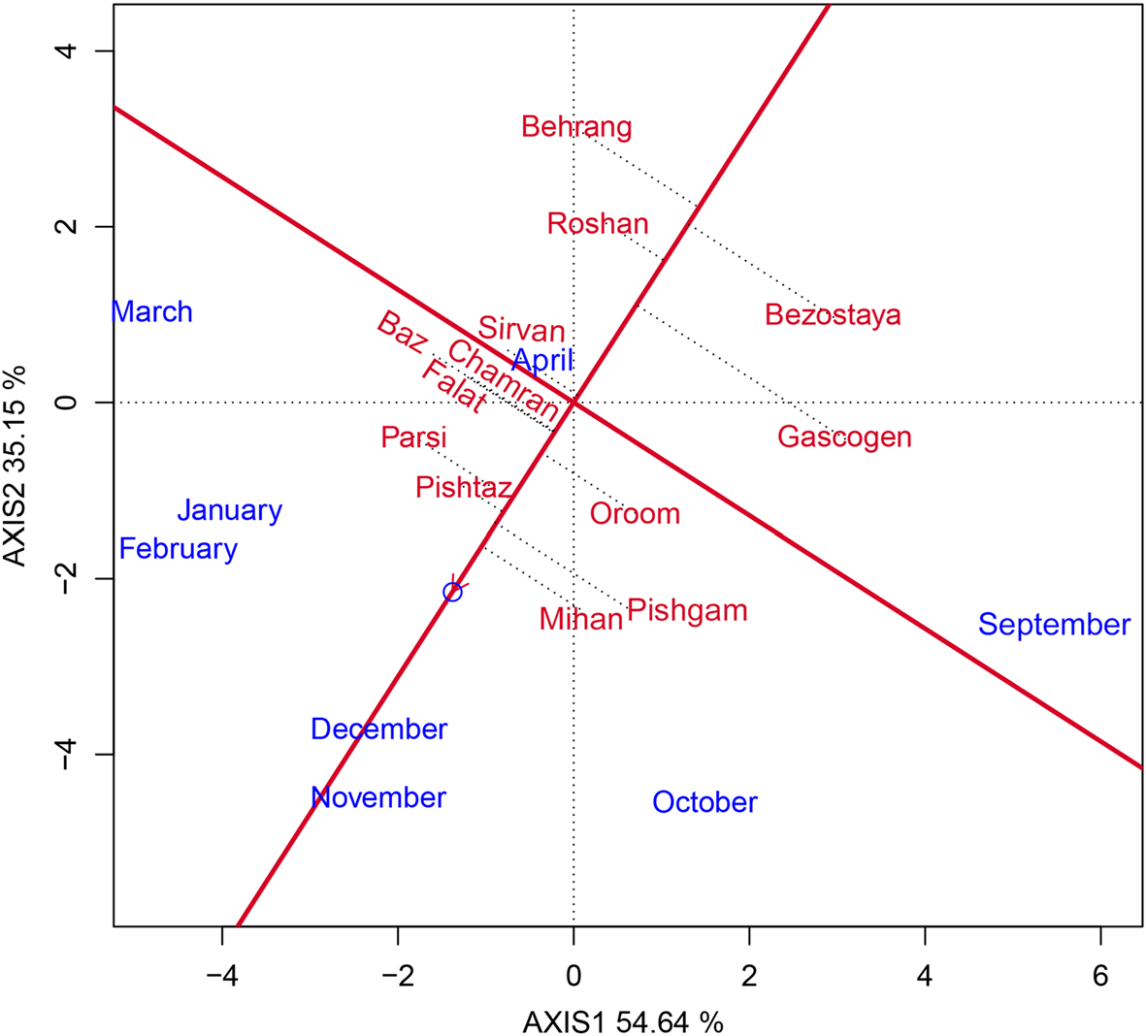
GGE biplot showing the ranking of wheat varieties based on grain yield performance and stability

**Fig. 3:**
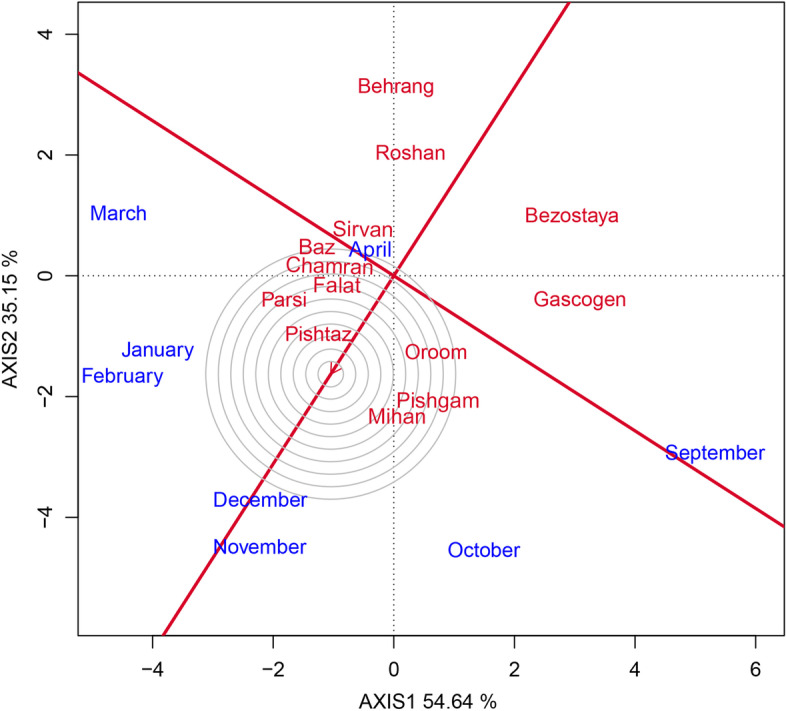
Comparison of wheat varieties against the position of an ‘ideal’ variety for grain yield and stability of performance across the sowing dates

**Fig. 4:**
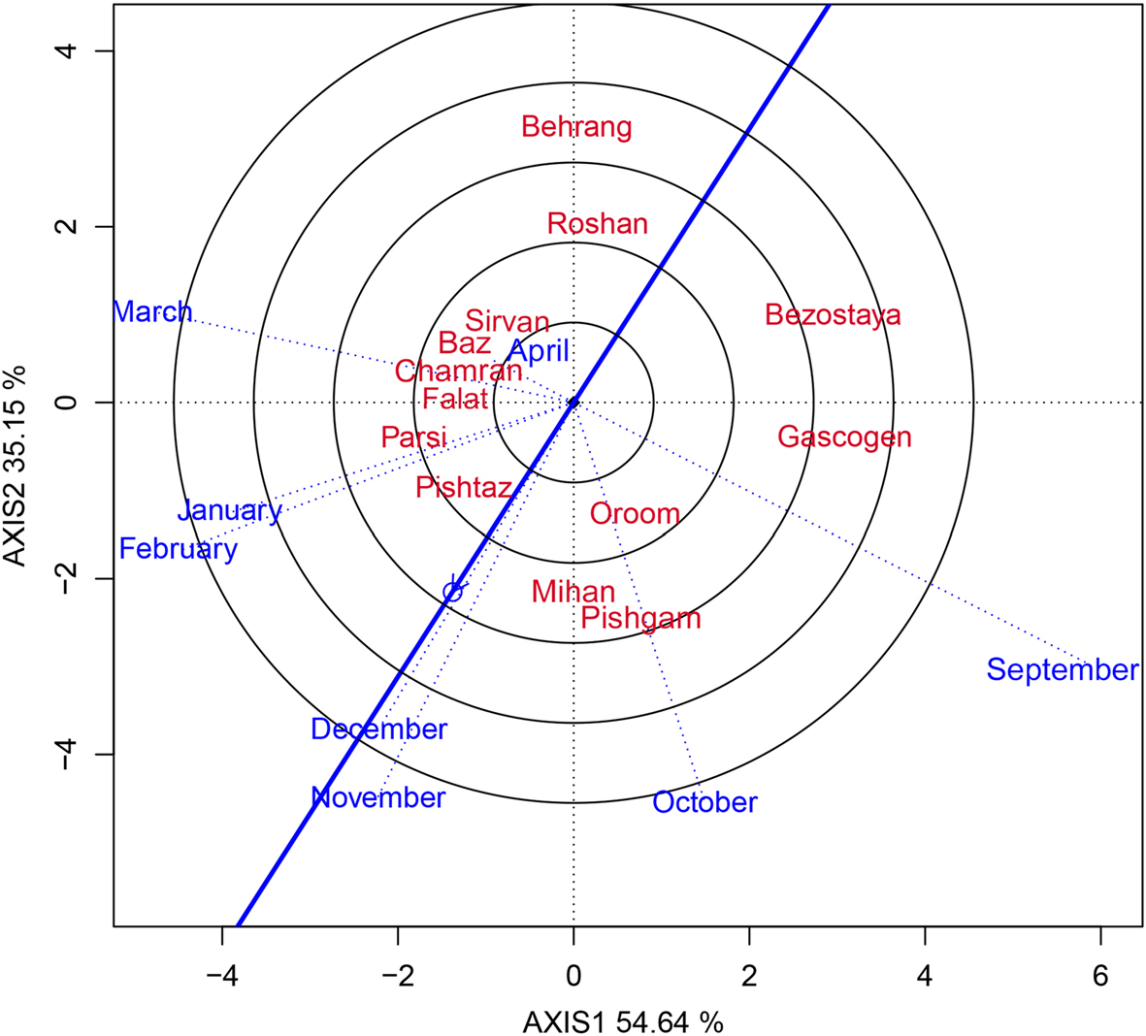
GGE biplot showing the discriminating power and representativeness of sowing dates

**Fig. 5:**
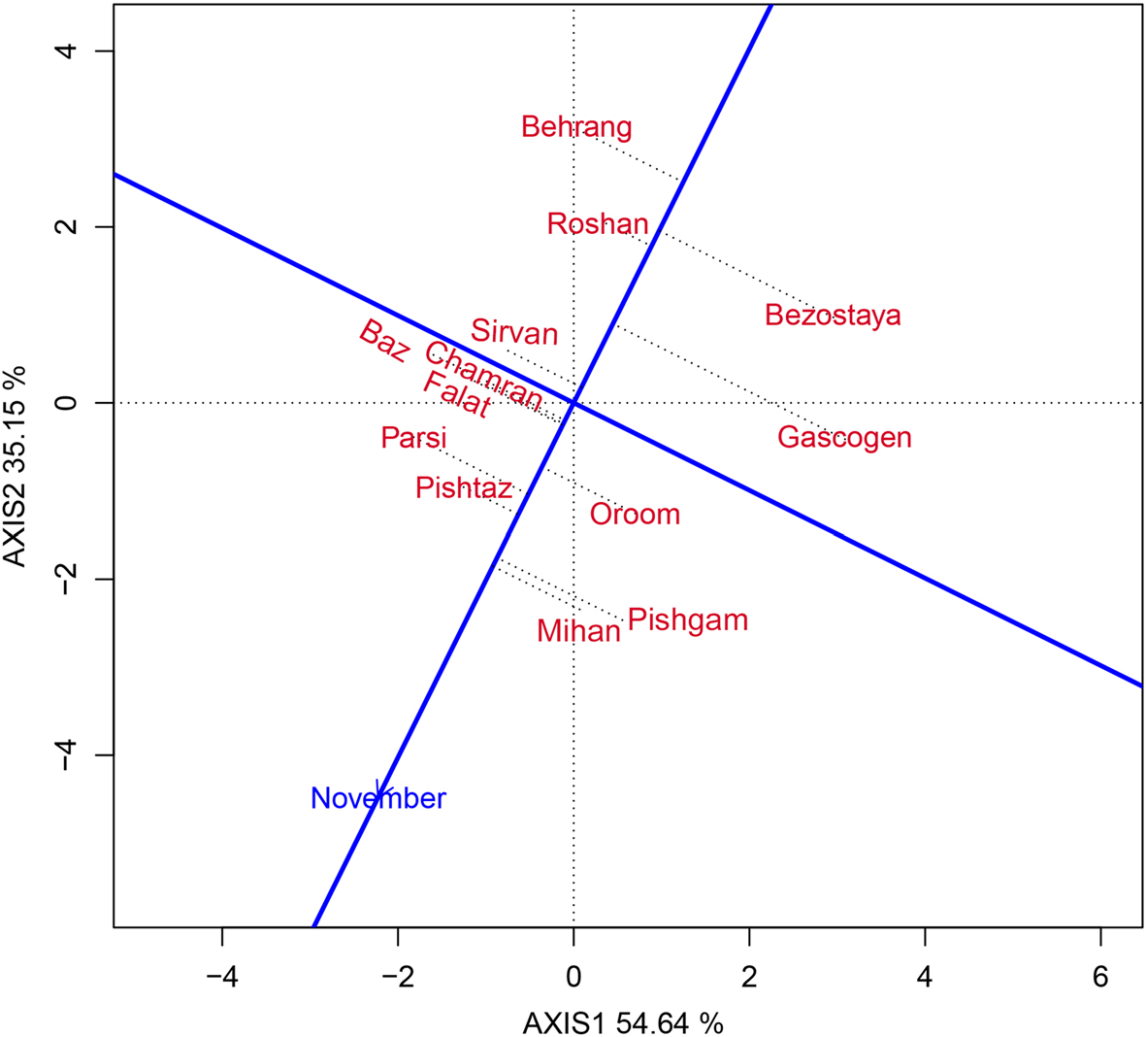
GGE biplot showing the performance of wheat varieties in an especial sowing date

In this study, the GEI effect was highly significant and justified 18.33% of the total variation of GY. As the existence of GEI complicates the selection process through reducing the usefulness of genotype via minimizing the association of genotypic and phenotypic values [27] therefore, the stability analysis is necessary. The environment (sowing date) effect was a major source of variation, which justified 72.21% of total variation; while, G and GE explained 6.94% and 18.33%, respectively (Table 6). This, along with the highly significant GEI, justified the use of stability analysis. Moreover, the large GE relative to G indicates the possible existence of different mega-environments with various top-yielding varieties [26]. The GGE biplot, which effectively determines the magnitude and pattern of GEI effect among the genotypes in a graphical manner, showed that the first two principal components (PCs) accounted for 54.64% and 35.15%, respectively, of the G + GE sum of squares, explaining a total of 89.79% of variation (Figs. 1, 2, 3, 4 and 5).

**Table 6:** Results of analysis of variance for grain yield of 13 wheat varieties evaluated at eight environments (sowing dates)

Source of variation	df	SS	MS	Total variation (%)
Total	311	2348.374		
Sowing date (S)	7	1695.752	242.250^**^	72.21
Variety (V)	12	162.867	13.572^**^	6.94
S × V	84	430.408	5.124^**^	18.33
PC1	18	303.328	16.852^**^	70.47
PC2	16	87.213	5.451^**^	20.26
PC3	14	18.683	1.335^**^	4.34
PC4	12	10.098	0.842^**^	2.35
PC5	10	7.041	0.704^**^	1.64
PC6	8	2.793	0.349^n.s^	0.65
PC7	6	1.252	0.209^n.s^	0.29
PC8	4	0.000	0.000^n.s^	0.00
Residual	208	59.280	0.285^n.s^	13.77
Error	297	2183.762	7.353	

^**^significant at 0.01 probability level

n.s: not significant

To effectively display the ‘‘which-won-where’’ pattern of the interaction between varieties and sowing dates and also to interpret their biplot for grain yield, the polygon view of the GGE biplot is given in Fig. 1. In the polygon biplot analysis, the varieties located on the vertices of the polygon (i.e. Behrang, Bezostaya, Gascogen, Pishgam, Parsi, and Baz) are the best or worst varieties on one or more sowing dates, because they have the most distances from the origin of biplot in their direction and hence, are considered specifically adapted. According to Fig. 1, Gascogen was the highest yielding variety on the sowing date of 6th September; Pishgam produced the highest yield on 6th October; Parsi was identified as the winner variety on 6th January, 6th February, and 6th March; and the variety of Baz showed high yield in 6th April; because these sowing dates were located in their related sections, respectively. Two vertex varieties of Behrang and Bezostaya, which fall in sections where there was no sowing date, are not the highest yielding in any sowing date; therefore, they were the poorest varieties in all or some of the sowing dates (Fig. 1).

The mean grain yield and stability performance of varieties are graphically displayed through the average environment coordination (AEC) method (Fig. 2). This method allows integration of grain yield and stability performance of varieties so as to identify the highest yielding and most stable varieties. In this figure, the single arrowhead line that passes through the origin of the biplot and marker for the average environment, and points towards higher mean values, is the AEC abscissa. The line perpendicular to the AEC, which passes through the origin of the biplot is referred to as the AEC ordinate, and is displayed as a double-arrowed line (Fig. 2). Projections of varieties on the AEC abscissa approximate the mean grain yield of them. The varieties farthest from the origin on the positive side of the AEC abscissa would have higher mean grain yield, and those farthest from the origin on the negative side of the AEC abscissa would have lower mean grain yield. Moreover, the greater the absolute length of the projection of a variety, the less stable it is [28]. According to Fig. 2, the varieties were divided into two groups. The first group, with above-average performance included: Mihan, Pishgam, Pishtaz, Parsi, Oroom, Chamran, Falat, and Baz, respectively. The stability ranking of the varieties in this group from most to least stable was: Pishtaz, Mihan, Parsi, Oroom, Chamran, Falat, Baz, and Pishgam. The second group that included the remaining varieties (Sirvan, Gascogen, Roshan, Bezostaya, and Behrang) had a below mean performance. A variety with the highest average performance of all varieties and absolutely stable in a wide range of environments is an ideal genotype [29, 30]. Therefore, considering both grain yield and stability performance, Pishtaz, Mihan, and Oroom were more stable as well as relatively high yielding in terms of grain yield. These varieties could be regarded as the most favorable ones (Fig. 2).

A unique feature of the GGE biplot is that it allows comparison of genotypes with the ideal genotype. In this regard, the genotypes which are located close to the ideal genotype in the biplot, are desirable ones. Thus, using the ideal variety as the center, concentric circles were drawn to help visualize the distance between each variety and the ideal variety. According to the ideal genotype view (Fig. 3), the variety of Pishtaz is positioned closest to the center of the concentric circles on the biplot, and therefore, could be considered as the most likely ideal variety. On the basis of GGE distance, the varieties of Mihan, Parsi, Pishgam, and Oroom were the closest to the ideal variety and could be regarded as desirable varieties. These varieties seem to be widely adapted to all sowing dates. On the other hand, the lower yielding varieties, i.e. Behrang, Roshan, Bezostaya, and Gascogen, are unfavorable ones, because they were located far away from the ideal variety, respectively (Fig. 3).

The discriminating power of the genotypes and representativeness of other environments, are two important features of an environment (Fig. 4). These features are important for the evaluation of environments in terms of their ability to effectively select the superior genotypes. The environments which have longer vectors are more discriminative and vice versa [23]. The representativeness of an environment is measured based on the angle between its vector and the ‘average environment coordinate’ (AEC) axis. Environments having a small angle with the AEC are more representative of other environments. An environment which has more discriminating power as well as is more representative of other environments is an ideal environment.

In this study, 6th November had the longest vector and therefore was the most discriminating sowing date, followed in order by 6th September, 6th October, 6th February, and 6th March (Fig. 4); however, 6th September, 6th October, and 6th March, which had wider angles with the AEC, were not representative of the other sowing dates and therefore, were not useful for selecting superior varieties. These sowing dates would be effective for the selection of varieties having specific adaptation. On the other hand, 6th April, with the shortest vector length, was the least discriminating sowing date; and the sowing date of 6th January was in the next rank. These sowing dates provide little or no information about the variability among varieties [31]. Sowing dates of 6th December, 6th November, 6th February, and 6th January, with the lower angles from the AEC axis, were the most representative sowing dates in order; and due to having the long vector length, were relatively high discriminative ones. Therefore, these sowing dates can be used to effectively select superior varieties, which can perform consistently best across all of the sowing dates. On the other hand, 6th April had the lowest discriminating power due to the short vector length, and was also the least representative environment, having the widest angle on the AEC axis. Therefore, 6th April cannot be used for the selection of superior varieties and is lower representative of the other sowing dates.

The GGE biplot can also be used for determining the relative performance of all genotypes in a specific environment. As an example, we consider the sowing date of 6th November, which had the highest grain yield among the other sowing dates. A line was drawn that passed through the biplot origin and the 6th November position to make an axis for this sowing date, and then a dashed line was perpendicularly drawn from each variety towards the 6th November axis (Fig. 5). The varieties were ranked based on their projections onto this axis, so that their rank increased in the direction towards the positive end [32]. In this example, the line that passed through the biplot origin and was perpendicular to the 6th November sowing date vector separated the varieties into two groups. The first group showed grain yields lower than average: Behrang, Bezostaya, Roshan, Gascogen, and Sirvan; and the second group had higher grain yields than average: Mihan, Pishgam, Pishtaz, Parsi, Oroom, Chamran, Falat, and Baz, respectively (Fig. 5).

## Discussion

Knowledge on the relative extent of genetic, environment, and their interaction effects is essential in the breeding of crop plants. The GGE biplot is a very powerful tool for the analysis and interpretation of GE; which effectively detects the interaction pattern graphically, identifies ‘which-won-where’ pattern, and delineates mega-environments among the test locations [14]. Combined analysis of variance indicated considerable genotypic variation for grain yield and its components, implying that there is high potential among the studied varieties for improving these traits through targeted selection in breeding programs, and was in agreement with the results of Gungor et al. [33] and Kendal [34]. The significant genotype × sowing date (GE) interaction demonstrated different genotypic response to environmental fluctuations, and the necessity of extension of analysis in multi-environment trails to calculate phenotypic stability. Similar results were also reported by Tekdal and Kendal [35] in durum wheat.

Wide genetic variation observed for all of the studied traits is promising for genetic progress through selection in the studied germplasm. More gain will result through selection when the difference between PCV and GCV is small, since it implies the more negligible effect of the environment and therefore higher heritability. In this study, smaller differences were observed between these coefficients for HI, BY, and TGW, indicating that more gain will result through selection for these traits. Jalata et al. [36] in barley showed that the difference between these two coefficients was smaller for phenological traits than for yield and its components.

Estimation of heritability is an important objective in breeding programs, because it determines the influence of environmental and genetic factors on the expression of the traits of interest, and provides early information for designing an effective breeding program to maximize genetic improvement [37]. In the present study, high heritability estimates were obtained for all traits with the exception of GY and GWSP, reflecting the presence of major genes or QTLs affecting these traits [38]. These results were generally consistent with the previous reports in wheat, suggesting major QTLs encoding for functional genes controlling most agronomic traits [39, 40]. Our estimates were higher than the moderate and low heritability estimates reported by Yagdi and Sozen [41] for several of the stated traits in a set of durum wheat under different environmental conditions. This confirms that, to some extent, the heritability estimates depend on the evaluated set of genotypes and target environments [42]. The most economically important trait of grain yield had a low heritability estimate, which led to a lower opportunity for improving this trait through phenotypic selection. Grain yield is a complex trait which is controlled by many minor genes; therefore, breeders often use indirect selection through strongly-correlated traits with grain yield, which have higher heritability estimates to improve it [43]. Several studies have attempted to estimate the heritability of important economic traits that directly affect yield response in wheat (e.g., [44]).

As the effect of yield components on yield is not explained by correlation coefficients [45], correlation path analysis was calculated to describe the direct and indirect associations between yield and a set of variables. Some studies reported that environmental changes can cause different responses to direct and indirect effects of variables on yield [46]. Results of the present study revealed that different sowing dates can change the associations of yield and its components, and this may be attributed to the amendment of direct and indirect effects of yield components. For example, under early sowing dates, NSP had the highest positive direct effect on grain yield, and therefore, was the most important and effective component of grain yield; while, under late ones, NGSP showed the highest positive direct effect on it and was the most important component of grain yield. The results showed that changes in the date of sowing wheat had amended the order of association among the components of grain yield. Therefore, indirect selection based on grain yield components in wheat can lead to different genetic gains in diverse sowing dates.

The GE interaction has been an important and challenging issue among plant breeders and agronomists involved in performance testing. This complicates the selection process, because it reduces the usefulness of genotypes by confounding their yield performance through minimizing the association of genotypic and phenotypic values [11]. Therefore, crop breeders are always seeking genotypes having high yield and low GEI. Assessment of genotypes in multiple environments would be effective for qualifying their performance. On the other hand, performing GE analysis improves the progress of genotype selection for cultivating in the interesting environment [47, 48]. In this study, the multivariate method of GGE biplot was applied to evaluate the stability of grain yield in wheat varieties grown at the different sowing dates.

As the AEC abscissa approximates the genotype contributions to G, the AEC ordinate must approximate the genotype contributions to GE, which is a quantification of stability or instability [17]. In this respect, Pishtaz, Mihan, and Oroom (the varieties having high and high grain yield) were also the most stable varieties, because they were located almost on the AEC abscissa and had a near-zero projection onto the AEC ordinate. This shows that their ranks were highly consistent across the sowing dates. By contrast, Gascogen, one of the high-yielding varieties, followed by Behrang, Bezostaya, and Pishgam, had the least stability and tended to be specifically adapted to certain sowing dates. Varieties of Pishtaz, Mihan, and Oroom had superior performance in all of the sowing dates, suggesting that they have broad adaptation to the diverse sowing dates.

Two groups of varieties were identified in terms of average performance. The first group, with above-average performance, included four varieties of Pishtaz, Mihan, Parsi, and Oroom, which were highly stable. The remaining varieties, i.e. Pishgam, Chamran, Falat, and Baz, showed moderate to low stability. The four varieties of Pishtaz, Mihan, Parsi, and Oroom, with high yield and highest stability, can be considered as the most desirable for different sowing dates, and using of them by farmers would result in stable performance across different sowing dates. However, Pishgam, Chamran, Falat, and Baz, which had high yield and low stability, are desirable for specific sowing dates. Overall, the varieties of the first group could also be used in the breeding programs for the development of new varieties with consistent performance. The second group, which had varieties with low yield but moderate to high stability, was considered as the more undesirable group.

According to the GGE biplot, an ideal genotype should have the highest mean performance of all genotypes and be absolutely stable to show broad adaptability in a wide range of environments [29]. In this regard, Pishtaz was recognized as the ideal variety, and four varieties of Mihan, Parsi, Pishgam, and Oroom were considered as the most desirable varieties, because they were located closest to the ideal variety. Among these five varieties, Pishtaz, Mihan, and Oroom, which had above-average performances and were located near the AEC abscissa and had short projections onto AEC ordinate (a near zero projection onto the mean– environment ordinate), could be considered as the most stable and optimal varieties. These varieties have wide adaptation to different sowing dates. In contrast, the other high-yielding varieties (Pishgam and Parsi) had lower stability, and hence, have specific adaptations to certain sowing dates.

## Conclusions

In conclusion, high genetic variation for agronomical studied traits showed the high potential of the studied germplasm for genetic improvement through targeted selection in breeding programs. The moderate to high broad-sense heritability for yield components (i.e. NSP, NGSP, GWSP, and TGW) showed that these traits are mainly under genetic effects; therefore, recurrent selection would be useful in achieving genetic advance for the above mentioned traits. However, the order of priority of these components and their direct and indirect effects was different for diverse sowing dates. So, under early sowing dates, NSP had the highest positive direct effect on the grain yield, and therefore, was the most important and effective component of grain yield; while, under late ones, NGSP showed the highest positive direct effect on it and was the most important component of grain yield. This suggests that indirect selection for the development of high yielding varieties should be done with a specific model. Moreover, because of the moderately low broad-sense heritability for grain yield, both genetic and non-genetic effects played a role in the genetic control of this trait. Therefore, selection based on an index may be more useful for the improvement of grain yield in recurrent selection programs. On the other hand, as the interaction of G × E is significant, selection of superior varieties for the improvement of wheat should be done based on the multi-environmental trails. From analysis of wheat varieties through the GGE biplot method, two groups of varieties were identified in terms of average performance. The first group, with above-average performance, included four varieties of Pishtaz, Mihan, Parsi, and Oroom, which were highly stable. The remaining varieties i.e. Pishgam, Chamran, Falat, and Baz showed moderate to low stability. Within the first group, three varieties of Pishtaz, Mihan, and Oroom had superior performance in all of the sowing dates, suggesting that they have broad adaptation to the diverse sowing dates. These varieties may be recommended for the genetic improvement of wheat with a high degree of adaptation. However, the varieties of the second group (i.e. Pishgam, Chamran, Falat, and Baz), which had high yield and low stability, are desirable for specific sowing dates.

## Methods

### Experimental site

This research was conducted for three years (2012—2014) at the research farm of the Mashhad Agricultural and Natural Resources Research Station, located in Torqabeh, Mashhad, Iran (2° 36′ N, 6° 59′ E, 1630 m amsl) on a silty loam soil, with pH 7.8. The mean annual precipitation and temperature of the region were 230 mm and 14.7^◦^C, respectively (www.havairan.com). According to the classification of Koppen, this region has a tropical and subtropical steppe cool climate. The monthly mean climatic variables (minimum temperature, maximum temperature, mean temperature, and rainfall) of this region over the years of this study are given in Table 7.

**Table 7:** Monthly temperature (˚c) and rainfall (mm) at the experimental site during 2012–15

Month	2012				2013				2014				2015			
	Rainfall	Temperature			Rainfall	Temperature			Rainfall	Temperature			Rainfall	Temperature		
		Min	Max	Mean		Min	Max	Mean		Min	Max	Mean		Min	Max	Mean
Jan	13.7	-0.8	11.2	5.2	25.6	1.8	15.1	8.5	6.5	-4	9.4	2.7	38.7	2.4	12.7	7.5
Feb	58.4	7.1	17.1	12.1	65.2	3.4	15.1	9.3	37.3	1.9	13.4	7.6	41.6	0.8	10.6	5.7
Mar	18	8.8	23.5	16.2	18.6	7.0	20.3	13.6	67.4	5.9	18.2	12.1	26.1	8.2	21.6	14.9
Apr	52.7	12.5	24.7	18.6	23.5	11.7	25.4	18.6	24.2	14.5	28.9	21.7	23.8	14.1	28.7	21.4
May	8.8	17.6	32.7	25.2	14.9	17.5	32.4	25	22	17.5	32.6	25.1	0.3	19	34.7	26.9
Jun	1.6	21.2	35.7	28.5	0	20.7	35	27.8	0	20.9	36.4	28.6	0	22.7	36.8	29.7
Jul	0	19.6	35.6	27.6	2.4	20.3	34.5	27.4	0	19.5	35.6	27.5	0	20.1	35.5	27.8
Aug	0	15.3	30.9	23.1	0	16.5	33.3	24.9	0	16.7	33	24.8	0.4	15.6	29.5	22.5
Sep	1.3	10.2	25.8	18	11.4	11.3	27.1	19.2	16.7	11.1	25.1	18.1	13.8	11.3	25.8	18.6
Oct	15.3	7.3	19.6	13.5	16.8	4.3	16.2	10.2	30.6	2.5	14.9	8.7	17.6	5.4	17	11.2
Nov	56.2	0.5	11.1	5.8	8.7	0.8	12.2	6.5	21.8	0.5	10.4	5.5	16	0.4	11.6	6
Dec	35	-4.8	8.1	1.7	0.6	-3.5	7.9	2.2	20.9	0.6	12.4	6.5	12.7	0.8	13.8	7.3

### Plant Materials and field evaluations

A set of thirteen wheat varieties, comprising of twelve hexaploid varieties of bread wheat, along with one tetraploid durum wheat (*T. turgidum*) variety were used as the genetic materials of this study (Table 8). The seeds of these varieties were kindly provided by the gene bank of Khorasan Razavi Agricultural and Natural Resources Research and Education Center. The experiment was laid out in a split-plot experiment according to a randomized complete block design with three replications, containing eight sowing dates viz. 6th September, 6th October, 6th November, 6th December, 6th January, 6th February, 6th March, and 6th April, as main plot treatments, and thirteen wheat varieties viz. Pishgam, Gascogen, Falat, Chamran, Roshan, Parsi, Pishtaz, Sirvan, Mihan, Oroom, Bezostaya, Behrang, and Baz, as subplot treatments during three years of study. In all three years, the seeds of each variety were sown in plots of four 300 cm long rows, 30 cm row spacing, and a within-row spacing of 10 cm. Cultural practices including irrigation, fertilization, and weed control were done each year, regularly. In order to keep the plots weed-free, weeds were controlled manually when necessary in all of the three years. Seeds were inoculated with fungicide before sowing and no further pest control program was implemented.

**Table 8:** Information of wheat varieties used in the study

Variety	Pedigree	Growth habit	Maturity status
Pishgam	*Bkt90-Zhong 87*	Facultative	Relatively early
Gascogen	*TJB-990–8/Marengo*	Winter	Medium
Falat	*(Kvz/Buho”s”//Kal/Bb)Seri 82*	Spring	Early
Chamran	*Attila, (CM85836-50Y-OM-OY-3 M-OY)*	Spring	Early
Roshan	*Selected from Iranian landrace*	Facultative	Early
Parsi	Dove”s”/Buc”s”/2*Darab	Spring	Early
Pishtaz	*Alvand//Aldan/Ias58*	Spring	Medium
Sirvan	PRL/2*PASTOR	Spring	Early
Mihan	*Bkt/90-Zhong87*	Winter	Medium
Oroom	*Alvand//NS732/Her*	Facultative	Medium
Bezostaya	*Lutescens-17/Skorospelka-3*	Winter	Late
Behrang	ZHONG ZUO/2*GREEN-3	Spring	Medium
Baz	WAXWING/4/SNI/TRAP-1/3/KAUZ,MEX*2/TRAP//KAUZ	Spring	Medium

The following agro-morphological characteristics were recorded during the period from 2012 to 2014. Thousand grain weight (TGW; g) was measured by counting three sub-sets of 100 grains randomly chosen from each variety and weighing them. Number of spikes (NSP), number of grains per spike (NGSP), and grain weight per spike (GWSP), were measured based on five randomly selected samples of each variety per replication, and the mean values of five plants were calculated and used for the analysis. Furthermore, assessments of grain yield (GY; t/ha) and biological yield (BY; t/ha) were done for each plot as a whole. The harvest index was calculated as the ratio of grain yield (GY) by the biological yield (BY), according to the following formula:

$$\mathrm{HI}\;(\%)\:=\:\mathrm{GY}/\mathrm{BY}\;\ast\;100$$

### Statistical analyses

Prior to analysis of variance (ANOVA), the normality distribution of data was initially checked by the Kolmogorov– Smirnov test, and the homogeneity of residual variance was examined by the Bartlett test, before combining the data of the three years. Subsequently, combined analysis of variance, proposed by Steel and Torrie [49], was performed to examine the differences between the varieties, sowing dates, years, and their interactions, and also to estimate the components of variance, using Proc GLM of SAS release 9.4 (SAS Institute, Cary, NC, USA). A split-plot experiment according to a randomized complete block design was used for the combined analysis of data obtained for three years on eight sowing dates, with sowing dates as the main plots and varieties as subplots. The effects of variety and sowing dates were considered as fixed effects, and the year was considered as random. Treatment mean comparisons were carried out using the Duncan's Multiple Range test at *p* ˂ 0.05. Components of variance were estimated by evaluating traits from mean squares of the ANOVA after being equated to their expected variance components [50]. Broad-sense heritability (h^2^_b_) was estimated on a phenotypic mean basis averaged over replications, years, and sowing dates (environments), according to Hallauer and Miranda [51]:

$${h}_{b}^{2}=\frac{{\sigma }_{g}^{2}}{{\sigma }_{g}^{2}+\frac{{\sigma }_{ge}^{2}}{e}+\frac{{\sigma }_{gy}^{2}}{y}+\frac{{\sigma }_{gey}^{2}}{ey}+\frac{{\sigma }_{\delta }^{2}}{re}+\frac{{\sigma }_{\epsilon }^{2}}{rey}}$$

where h^2^_b_ is the broad-sense heritability, ${\sigma }_{g}^{2}$ is the genotype, ${\sigma }_{ge}^{2}$ is the genotype × environment, ${\sigma }_{gy}^{2}$ is the genotype × year, ${\sigma }_{gey}^{2}$ is the genotype × environment × year variance; ${\sigma }_{\delta }^{2}$ and ${\sigma }_{\epsilon }^{2}$ are the error variance and the residual variance, respectively; while, g, e, y, and r represent the number of genotypes (varieties), environments (sowing dates), years, and replications, respectively. To estimate the level of genetic variation, the phenotypic coefficient of variation (PCV) and genetic coefficient of variation (GCV) were calculated using the following equation:

$$\begin{array}{c}\mathrm{PCV }= (\mathrm{\sigma p }/\upmu ) 100\\ \mathrm{GCV }= (\mathrm{\sigma g }/\upmu ) 100\end{array}$$

where σ_p_ is the square root of the phenotypic variance, σ_g_ is the square root of the genotypic variance, and µ is the phenotypic mean. Stepwise multiple linear regression analysis was used to determine the variables accounting for the majority of grain yield variability. Path coefficient analysis was conducted on the basis of phenotypic correlation coefficients, taking grain yield as an effect and the characters that were added to the multiple linear regression models as a cause.

### Stability analysis

For the stability analysis, each sowing date was considered as the test environment, creating eight test environments. After verifying the existence of significant GE interaction, analysis of adaptability and grain yield stability was performed by the GGE biplot analysis, using the model described by Yan [52]. To identify stable and adaptive varieties using the GGE biplot, data were graphically analyzed for interpreting the GE interaction [14]. For this purpose, the first two components resulting from singular value decomposition (SVD), were used to draw the biplots using GEA-R version 4.1. [53]. The remaining PCs were regarded as residuals [14].

## Data Availability

All data generated or analyzed during this study are included in this published article.

## References

[1] Ye X, Li J, Cheng Y, Yao F, Long L, Wang Y, Wu Y, Li J, Wang J, Jiang Q (2021). Genome-wide association study reveals new loci for yield-related traits in Sichuan wheat germplasm under stripe rust stress. BMC Genom.

[2] Bilgrami SS, Darzi Ramandi H, Shariati V, Razavi Kh, Tavakol E, Fakheri BA, Mahdi Nezhad N, Ghaderian M (2020). Detection of genomic regions associated with tiller number in Iranian bread wheat under different water regimes using genome-wide association study. Sci Rep.

[3] Atchison J, Head L (2010). Wheat as food, wheat as industrial substance: comparative geographies of transformation and mobility. Geoforum.

[4] Crespo-Herrera LA, Crossa J, Huerta-Espino J, Vargas M, Mondal S, Velu G, Payne TS, Braun H, Singh RP (2018). Genetic gains for grain yield in CIMMYT's semi-arid wheat yield trials grown in suboptimal environments. Crop Sci.

[5] Khichar ML, Niwas R (2006). Microclimatic profiles under different sowing environments in wheat. J Agrometeorol.

[6] Gibson LR, Schwarte AJ, Sundberg D, Douglas LK. Planting date effects on winter triticale grain yield. Iowa state university, ISRF04–12; 2007.

[7] Hossain I, Epplin FM, Krenzer JEG (2003). Planting date influence on dual-purpose winter wheat forage yield, grain yield, and test weight. Agron J.

[8] Flowers M, James C, Petrie S, Machado S, Rhinhart K (2006). Planting date and seeding rate effects on the yield of winter and spring wheat varieties results from the 2005–2006 cropping year. Agri Res.

[9] Subhan F, Khan M, Jamro GH (2004). Effect of different planting date, seeding rate and weed control method on grain yield and yield components in wheat. Sharhad J Agr.

[10] Saeidnia F, Majidi MM, Mirlohi A (2017). Genetic analysis of stability in poly-crossed populations of orchardgrass. Crop Sci.

[11] Bantayehu M (2010). Analysis and correlation of stability parameters in malting barley. Afr J Crop Sci.

[12] Oral E, Kendal E, Dogan Y (2018). Selection the best barley genotypes to multi and special environments by AMMI and GGE biplot models. Fresenius Environ Bull.

[13] Amini F, Majidi MM, Mirlohi A (2013). Genetic and genotype × environment interaction analysis for agronomical and some morphological traits in half-sib families of tall fescue. Crop Sci.

[14] Yan W, Tinker NA (2006). Biplot analysis of multi-environment trial data: principles and applications. Can J Plant Sci.

[15] Eberhart SA, Russell WA (1966). Stability parameters for comparing varieties. Crop Sci.

[16] Zobel RW, Wright MJ, Gauch HG (1988). Statistical analysis of a yield trial. Agron J.

[17] Yan W (2001). GGE biplot-A windows application for graphical analysis of multi-environment trial data and other types of two-way data. Agron J.

[18] Jandong EA, Uguru MI, Oyiga BC (2011). Determination of yield stability of seven soybean (*Glycine max*) genotypes across diverse soil pH levels using GGE biplot analysis. J Appl Biosci.

[19] Zhang PP, Song H, Xi-Wang K, Xi-Jun J, Li-hua Y, Yang L, Yang QU, Wang SU (2016). GGE biplot analysis of yield stability and test location representativeness in proso millet (*Panicum miliaceum* L.) genotypes. J Integr Agric..

[20] Teodoro PE, Almeida Filho JE, Daher RF, Menezes CB, Cardoso MJ, Godinho VPC, Torres FE, Tardin FD (2015). Identification of sorghum hybrids with high phenotypic stability using GGE biplot methodology. Genet Mol Res.

[21] Gauch HG, Piepho HP, Annicchiarico P (2008). Statistical analysis of yield trials by AMMI and GGE: Further considerations. Crop Sci.

[22] Yan W, Hunt LA, Sheng Q, Szlavnics Z (2000). Cultivar evaluation and mega-environment investigation based on the GGE biplot. Crop Sci.

[23] Yan W, Kang MS, Ma B, Woods S, Cornelius PL (2007). GGE biplot vs. AMMI analysis of genotype-by-environment data. Crop Sci..

[24] Hao ZF, Li XH, Su ZJ, Xie CX, Li MS, Liang XL, Weng JF, Zhang DG, Li L, Zhang S (2011). A proposed selection criterion for drought resistance across multiple environments in maize. Breed Sci.

[25] Leilah AA, Al-Khateeb SA (2005). Statistical analysis of wheat yield under drought conditions. J Arid Environ.

[26] Saeidnia F, Majidi MM, Mirlohi A, Dehghani MR, Hosseini B (2021). Yield stability of contrasting orchardgrass (*Dactylis glomerata* L.) genotypes over the years and water regimes. Euphytica.

[27] Crossa J (1990). Statistical analysis of multi-location trials. Adv Agron.

[28] Kaya Y, Ak¸cura M, Taner S (2006). GGE-biplot analysis of multi-environment yield trials in bread wheat. Turk J Agric For.

[29] Akc¸ura M, Taner S, Kaya Y (2011). Evaluation of bread wheat genotypes under irrigated multi-environment conditions using GGE biplot analyses. Agriculture.

[30] Sharma RC, Morgounov AI, Braun HJ, Akin B, Keser M, Bedoshvili D, Bagci A, Martius C, Van Ginkel M (2010). Identifying high yielding stable winter wheat genotypes for irrigated environments in Central and West Asia. Euphytica.

[31] Jalata Z (2011). GGE-biplot analysis of multi-environment yield trials of barley (*Hordeum vulgare* L.) genotypes in Southeastern Ethiopia Highlands. Int J Plant Breed Genet..

[32] Yan W, Hunt LA (2002). Biplot analysis of diallel data. Crop Sci.

[33] Gungor H, Cakir MF, dumlupinar Z. Evaluation of some advanced bread wheat (*Triticum aestivum* L.) lines for agronomic traits under Kirhlareli and Tekirdag conditions. Black Sea J of Agric. 2022. 10.47115/bsagriculture.1074104.

[34] Kendal E. Comparing durum wheat cultivars by genotype × yield × trait and genotype × trait biplot method. Chil. J. Agric. Res. 2019; 79(4). 10.4067/S0718-58392019000400512.

[35] Tekdal S, Kendal E (2018). AMMI model to assess durum wheat genotypes in multi-environment trials. J Agr Sci Tech.

[36] Jalata Z, Ayana A, Zeleke H (2011). Variability, heritability and genetic advance for some yield and yield related traits in Ethiopian barley (*Hodeum vulgare* L.) landraces and crosses. Int J Plant Breed Genet..

[37] Hallauer AR, Carena MJ, Miranda JB (2010). Quantitative genetics in maize breeding.

[38] Mwadzingeni L, Shimelis H, Tsilo TJ (2017). Variance components and heritability of yield and yield components of wheat under drought-stressed and non-stressed conditions. Aust J Crop Sci.

[39] Li X, Xia X, Xiao Y, He Z, Wang D, Trethowan R, Wang H, Chen X (2015). QTL mapping for plant height and yield components in common wheat under water-limited and full irrigation environments. Crop Pasture Sci.

[40] Mathews KL, Malosetti M, Chapman S, McIntyre L, Reynolds M, Shorter R, Eeuwijk FV (2008). Multi-environment QTL mixed models for drought stress adaptation in wheat. Theor Appl Genet.

[41] Yagdi K, Sozen E (2009). Heritability, variance components and correlations of yield and quality traits in durum wheat (*Triticum durum* Desf.). Pak J Bot..

[42] Eid MH (2009). Estimation of heritability and genetic advance of yield traits in wheat (*Triticum aestivum* L.) under drought condition. Int J Genet Mol Biol..

[43] Sallam A, Hamed ES, Hashad M, Omara M (2014). Inheritance of stem diameter and its relationship to heat and drought tolerance in wheat (*Triticum aestivum* L.). J Plant Breed Crop Sci..

[44] Abdolshahi R, Nazari M, Safarian A, Sadathossini T, Salarpour M, Amiri H (2015). Integrated selection criteria for drought tolerance in wheat (*Triticum aestivum* L.) breeding programs using discriminant analysis. Field Crops Res..

[45] Saeidnia F, Majidi MM, Mirlohi A, Manafi M (2017). Productivity, persistence and traits related to drought tolerance in smooth bromegrass. Plant Breed.

[46] Talebi R, Fayyaz F, Mohammad-naji A (2010). Genetic variation and interrelationships of agronomic characteristics in durum wheat under two constructing water regimes. Braz Arch Biol Technol.

[47] Hassani M, Heidari B, Dadkhodaie A, Stevanato P (2018). Genotype by environment interaction components underlying variations in root, sugar and white sugar yield in sugar beet (*Beta vulgaris* L.). Euphytica.

[48] Kendal E, Sener O (2015). Examination of genotype × environment interactions by GGE biplot analysis in spring durum wheat. Indian J Genet.

[49] Steel RGD, Torrie JG (1980). Principles and procedures of statistics.

[50] Nguyen HT, Sleper DA (1983). Theory and application of half-sib matings in forage breeding. Theor Appl Genet.

[51] Hallauer AR, Miranda JB (1988). Quantitative genetics and maize breeding.

[52] Yan W (2002). Singular-value partitioning in biplot analysis of multi-environment trial data. Agron J.

[53] Pacheco A, Vargas M, Alvarado G, Rodríguez F, Crossa J, Burgueño J. "GEA-R (Genotype x Environment Analysis with R for Windows) Version 4.1", CIMMYT Research Data & Software Repository Network, V16; 2015 https://hdl.handle.net/11529/10203.

